# GenomicLayers: sequence-based simulation of epi-genomes

**DOI:** 10.1186/s12859-025-06224-y

**Published:** 2025-08-04

**Authors:** Dave T. Gerrard

**Affiliations:** Division of Evolution, Infection and Genomics, School of Biological Sciences, Faculty of Biology, Medicine & Health, The University of Manchester, Stopford Building, Oxford Road, Manchester, M13 9PT UK

**Keywords:** Epigenome, Simulation, R, Genome, Development

## Abstract

**Background:**

Cellular development and differentiation in Eukaryotes depends upon sequential gene regulatory decisions that allow a single genome to encode many hundreds of distinct cellular phenotypes. Decisions are stored in the regulatory state of each cell, an important part of which is the epi-genome—the collection of proteins, RNA and their specific associations with the genome. Additionally, further cellular responses are, in part, determined by this regulatory state. To date, models of regulatory state have failed to include the contingency of incoming regulatory signals on the current epi-genetic state and none have done so at the whole-genome level.

**Results:**

Here we introduce GenomicLayers, a new R package to run rules-based simulations of epigenetic state changes genome-wide in Eukaryotes. Simulations model the accumulation of changes to genome-wide *layers* by user-specified *binding factors*. As a first exemplar, we show two versions of a simple model of the recruitment and spreading of epigenetic marks near telomeres in the yeast *Saccharomyces cerevisiae*. By combining the output from 100 runs of the simulation, we generate whole genome predictions of epigenetic state at 1 bp resolution. The example yeast models are included within a ‘vignette’ with the GenomicLayers package, which is available at https://github.com/davetgerrard/GenomicLayers. To demonstrate the use of GenomicLayers on the full human reference genome (hg38), we show the results from parameter refinement on a simplistic model of the action of pluripotency factors against a self-spreading repressor seeded at CpG islands. The human genome model is included in supplementary information as an R script.

**Conclusions:**

GenomicLayers enables scientists working on diverse eukaryotic organisms to test models of gene regulation in silico. Applications include epigenetic silencing, activation by combinatorial binding of transcription factors and the sink effects caused by down-regulation of components of epigenetic regulators. The software is intended to be used to parameterise, refine and combine models and thereby capitalise on data from the thousands of studies of Eukaryotic epigenomes.

**Supplementary Information:**

The online version contains supplementary material available at 10.1186/s12859-025-06224-y.

## Background

In Eukaryotes, a single genome can encode diverse phenotypes, or cell types, by integrating environmental cues, cell-to-cell communication and internal regulatory networks. Such cell states can be retained through cell division by epigenetic memory (the epigenome) to direct daughter cells to appropriate phenotypes and this process is essential for development and differentiation in many multi-cellular organisms [1, 5, 8, 13, 27, 37, 38]. In turn, the epigenome may influence further cell fate decisions such that a single signal can lead to alternate outputs from different cell types. For example, some transcription factors bind to and activate discreet subsets of genomic targets governed by the local state of chromatin [22]. Therefore, a complete model of the link between genotype and phenotype needs to include changes to regulatory state that recapitulate those that happen during development.

Furthermore, it is important to be able to include the whole genome in such models because epigenomic regulation may be susceptible to “sink effects”, whereby activity of epigenetic machinery over one part of the genome alters the availability of the same machinery to act elsewhere. For example, in human embryonic stem cells directed to a neuronal cell fate, knockdown of the DNA-methyltransferase DNMT3B, which methylates CpG di-nucleotides, causes redirection of the histone modification H3K27me3 to regions that would otherwise be marked with DNA methylation [21]. Even without perturbation, spreading of epigenetic states likely plays a significant role in the establishment of expressed and non-expressed regions, with boundary positions determined by the relative rates of spreading from regulatory elements [36]. Sink effects can be local or seen at the whole genome level. In Drosophila, the presence of extra Y chromosomes, which recruit a high proportion of the repressive machinery in the nucleus, cause depletion of repression across the rest of the genome and result in aberrant activation of transposable elements and some genes [3]. Together, these properties of epigenomic regulation suggest that models that do not include a sequential or developmental component including feedback between epigenomic states and regulatory decisions, will be unable to replicate the patterns seen in living cells.

There are already myriad experimental data on the functions of individual epigenetically active biomolecules and extensive databases of epigenomic states from diverse cell types and diseases [29]. However, we lack software to explicitly test our models of how specific transcription factors and various writers and erasers of the epigenome interact over time to influence development. Previous modelling has either ignored the DNA sequence (e.g. [6]) or have operated only over individual loci (e.g. [25]). Here, I introduce GenomicLayers as an environment in which to implement research-inspired models at whole genome scale and test them against experimental data or against each other.

## Implementation

GenomicLayers is implemented as an R package and can be installed directly from https://github.com/davetgerrard/GenomicLayers. GenomicLayers simulations have been created on a selection of eukaryotic haploid genome sequences of varying size from *Saccharomyces cerevisiae* (SacCer3, ~ 12 Mbp) to human (hg38, ~ 3.2 Gbp).

**Fig. 1:**
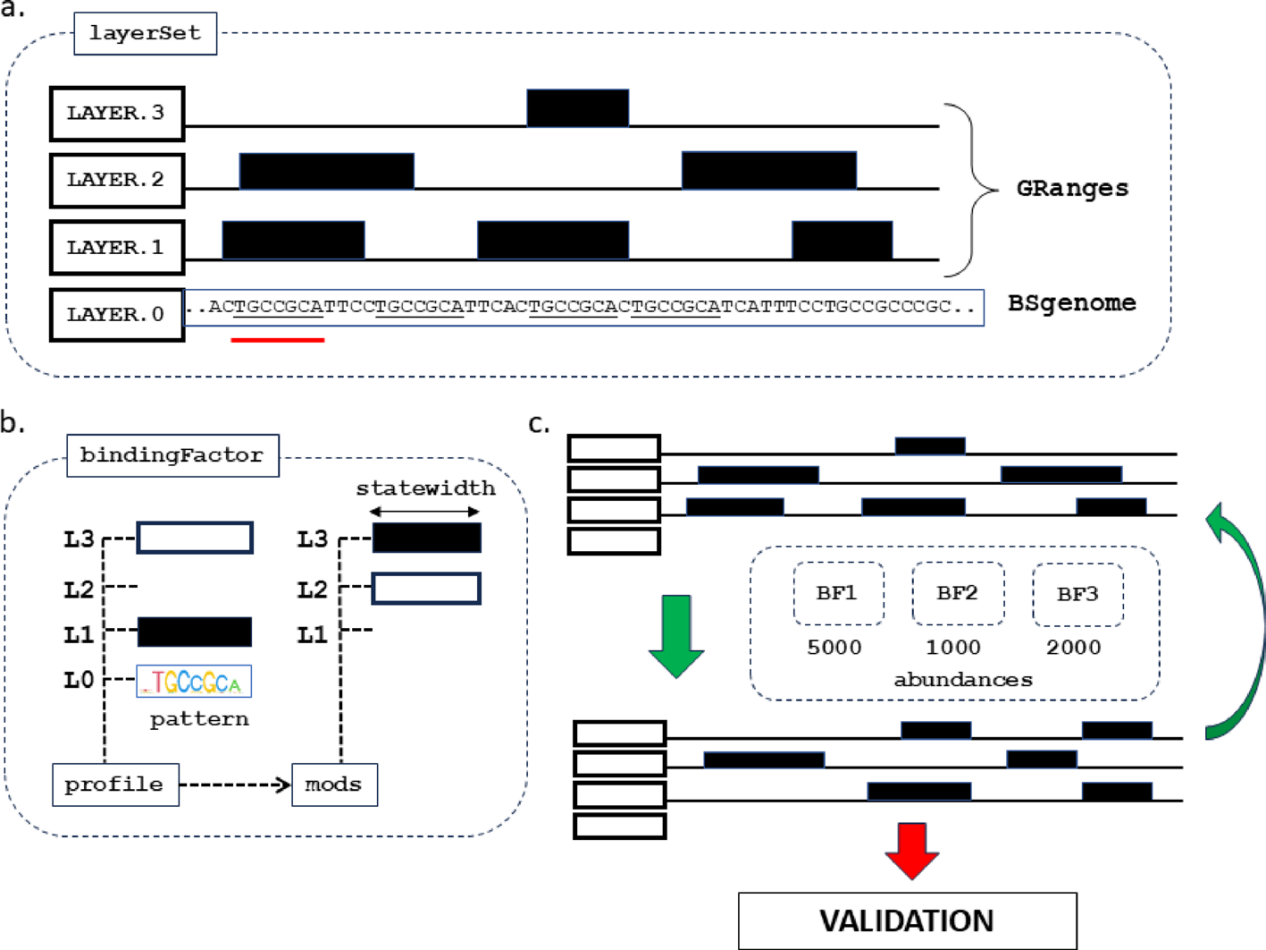
Core components of the GenomicLayers package. **a** Each simulation requires one ‘*layerSet’* comprising a genome sequence as a *BSgenome* object and one or more layers as *GRanges* objects. The *GRanges* ‘layers’ are modified during the simulation and can be arbitrarily named. **b** One or more ‘*bindingFactors’* each contain a ‘profile’ to match with combinations of layer states and/or the genome sequence. Sequence matches can be represented either as consensus sequences (using IUPAC codes), as position weight matrices (shown here as a seqLogo), or as regular expressions. The profile shown here only fully matches (both sequence and layers) at the position in a) shown by the red line (e.g. a sequence match AND LAYER.1 = present AND LAYER.3 = absent; LAYER.2 is ignored). Once a set of matches have been selected, the ‘mods’ of a binding factor dictate changes to the layerSet at that position (e.g. LAYER.2 set to absent AND LAYER.3 set to present). The extent of the modifications is determined by the statewidth parameter, which is independent of the width of the matching pattern. **c** A simulation comprises one or more cycles during which one or more bindingFactors are matched to the current state of the layerSet. The number of modifications performed by each bindingFactor each cycle is limited by a vector of ‘abundance’ values, which may be kept constant or varied between cycles. The layerSet state after modification becomes the starting state for the next cycle (green arrows) or can be measured against validation data (red arrow)

Simulations in GenomicLayers require two primary components: firstly, a *'layerSet'* comprising a textual representation of the whole genome (the genome sequence) linked to any number of ‘layers’ to record genomic states (Fig. 1a); secondly, a set of ‘*bindingFactor’* (BF) objects, representing either specific molecules that act upon the layers or auxiliary objects that help implement regulatory logic (Fig. 1b). Simulations are lightweight and ‘rules-based’ and do not attempt to simulate physical or chemical properties of components.

GenomicLayers builds on existing and widely used R packages available in the Bioconductor repository. The genomic sequence in the layerSet is a *BSgenome* object [26]. The genomes of model organisms (e.g. SacCer3, mm10, hg38) can be installed directly into R from their respective package repositories. Alternatively, *BSgenome* objects can be generated from any set of *fasta* sequences using the BSgenome package. This enables use of genome sequence from non-model organisms, from individuals, and potentially from in silico mutagenesis experiments. The ‘layers’ in the *layerSet* object are *GRanges* objects [18]. By default, these begin as empty *GRanges* objects but can be initiated with a set of ranges or loaded from a previous simulation. The *layerSet* may also store a cache of sequence hits for some of the *bindingFactors* to reduce the need to repeat searches where it is expected the results will not change between cycles (e.g. potential binding sites of DNA-binding transcription factors). Finally, the *layerSet* includes a history table to store simple statistics about each layer after each cycle.

The second component type, ‘*BindingFactors’*, each comprise a ‘*profile’* that matches one or more layers matching the layerSet and a set of ‘*modifications’* that represent the changes to be made to the layerSet where a binding factor finds a profile match (Fig. 1b). Profile matches to the genome sequence can be specified as simple text (including IUPAC codes), as position weight matrices, or as grep-style ‘regular expressions’. Profile matches to the other non-genome layers are binary and represent presence/absence of a mark and can be combined with AND or NOT. Modifications can be applied as present/absent to any or all of the layers for each binding factor. The genome sequence cannot be modified. The width of profile matches and modifications to layers are controlled by separate length parameters so that binding factors can change the state in an area larger (or smaller) than the length of their binding pattern. Using R’s functional programming, it is possible to pass functions to some parameters and generate values dynamically from a defined distribution.

A simulation comprises one or more cycles during which all the binding factors operate on and update the regulatory state(s) stored in the layerSet (Fig. 1c). The number of changes per cycle is controlled by a vector of abundances representing the relative activity of each factor. Abundances can be fixed for the entire simulation or be dynamically recalculated between cycles allowing for feedback from a gene-regulatory network (not implemented here). The output of the model is the modified layerSet object which contains a new set of GRanges for some, or all, of the layers. These GRanges objects can be used directly in many other Bioconductor packages (e.g. to measure concordance with experimental data), exported as tables (e.g. as bed files) or saved as R objects and re-loaded to initiate a layerSet for further simulations. Rapid methods already exist to calculate genome coverage across multiple results (e.g. the GenomicRanges function coverage()), which means that the average signal of many repeated simulations can easily be calculated (see Results).

## Results

### Model example A: accumulation of repressive SIR proteins at telomeres in budding yeast, *Saccharomyces cerevisiae*

In yeast, the silent information regulator (SIR) complex of proteins accumulate at specific locations on chromosomes to form heterochromatin and silence transcription [30]. During logarithmic growth, most SIR-related heterochromatin is restricted to telomeres whereas during stationary phase, SIR heterochromatin also forms intra-chromosomally to reduce transcriptional output [34]. SIR is thought to be recruited to telomeres by the DNA-binding factor RAP1, which binds to the sequence GGTGT [16, 20, 35]. RAP1 has two very similar DNA-binding domains and may prefer adjacent, repeated motifs [20, 35]. Once recruited, the SIR complex are capable of recruiting further copies of themselves causing the repressive state to spread along the chromosome [30]. Several other features of this system are known [15, 30], but here I model only the recruitment and spreading.

I generated two models using the *S. cerevisiae* (SacCer3) genome. In Model 1, bindingFactor RAP1 binds to a single GGTGT motif and marks a layer “sir3_potential”, which then serves as a recruitment target for bindingFactor ‘Sir3p’ (representing the SIR complex). Once Sir3p is recruited, it marks a layer “sir3_bound” which serves to recruit a further bindingFactor, ‘Sir3.spreader’, which adds more ranges to the sir3_bound layer in the vicinity of a match. The distance and direction of the spreading is controlled by a function that dynamically generates normally distributed distances (mean = 147, standard deviation = 30). Model 2 is the same as Model 1 except that RAP1 will only be recruited to double motifs gapped by up to 3 bp. Models were run for 200 cycles with layers saved to disk every 10 cycles. Each simulation was repeated 100 times using a high-performance compute cluster at the University of Manchester. Both versions of the model are included with the package as a vignette (‘sacCer3_rap1_B2_nucGenome’).

**Fig. 2:**
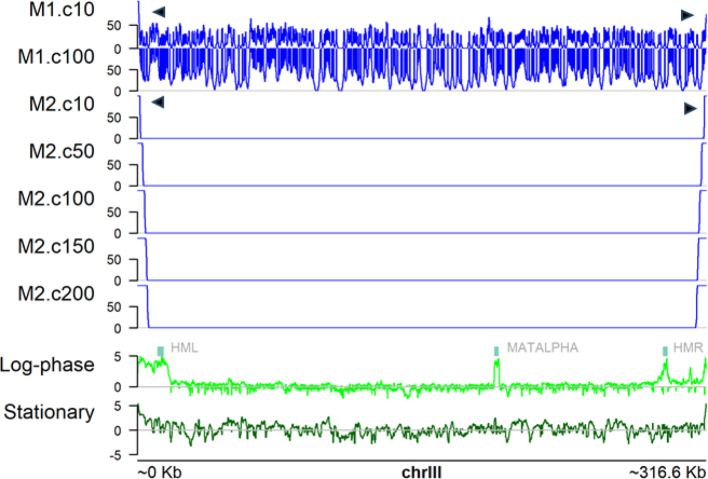
Output of whole genome Sir3 recruitment and spreading models M1 and M2 on *Saccharomyces cerevisiae* chromosome III (SacCer3: chrIII). Output from 100 replicated simulations were stored every 10 cycles (e.g. M2.c50 = Model 2, cycle 50). The outputs were summed using the function coverage() (GenomicRanges package) representing the number of simulations in which each base of the chromosome was marked by Sir3p. Note the high frequency of early Sir3p recruitment at the very tips of the telomeres in both models (denoted by filled arrowheads). Model 1, which allows recruitment of Sir3p from monomeric Rap1 binding, generates Sir3p binding all across the chromosome. Model 2 requires two adjacent Rap1 sites to seed Sir3p binding and has fewer seed sites from which Sir3p binding spreads out along the chromosome. The bottom two plots show Sir3p binding signal for Log-phase and stationary phase samples (light and dark green, respectively) measured using chromatin immunoprecipitation on a genome-wide tiling array (ChIP-chip) [34]. ‘MATALPHA’ marks the mating type-locus, which is repressed by Sir3p binding in the log-phase sample. Plot generated using plotSignal function (plotGardener package [17])

Figure 2 shows the summed coverage along SacCer3 chrIII of 100 replicate simulations for both models (in blue) alongside tiling array ChIP signal of Sir3p in both logarithmic growth and stationary phases [34]. All 16 SacCer3 chromosomes are shown in supplemental data 1. Modelling recruitment of SIR to a single RAP1 motif (Fig. 2, Model 1) fails to restrict accumulation of Sir3p at the telomeres. There are initial high levels of binding at the very tips of the telomeres by cycle 10 (Fig. 2, black triangles), but intra-chromosomal seeding events at single motifs cause Sir3p to be recruited chromosome wide.

In contrast, when seeding of Sir3p is constrained to only occur at two motifs gapped by 0–3 bases (Fig. 2, Model 2), there is a much stronger bias toward the telomeres where more double motifs occur. Subsequent spreading then more closely resembles the experimental data at the p-arm of the chromosome. As expected, the model does not generate a peak over the mating type locus (MATALPHA in Fig. 2) as epigenetic silencing of this region is reversible independent of growth phase and unlikely to be directly determined by sequence [23]. The results are similar for most chromosomes (supplemental Fig. 1) with varying degrees of agreement. For several chromosomes, Model 2 also generates artefactual peaks at intra-chromosomal locations corresponding to double RAP1 motifs. These models could easily be extended to include more known features of heterochromatin formation during log-phase and further extended to model the re-distribution of SIR proteins during Stationary phase [31].

### Model example B: ‘activation’ of promoters in the human genome against a background CpG island repressor

In many vertebrates, including humans, the expression of combinations of specific transcription factors (TFs) early in development (a.k.a. Yamanaka factors) are required to activate the developmental cascade and can be experimentally induced to re-programme differentiated cells into pluripotent stem cells (iPSCs)[32]. The human TFs OCT4 (POU5F1), SOX2, KLF4, and MYC (collectively: OSKM) are able to bind to enhancers of epigenetically repressed genes and re-activate transcription at nearby gene promoters [32]. A variety of mechanisms have been proposed to explain the precedence and interaction of the OSKM factors and their interactions with other epigenetic states [2, 4, 9, 19]. Therefore, it may be of use to the community to explore such models with simulations. Here, I describe a basic model and show how it can be used to explore parameter choices.

I retrieved consensus DNA binding motifs for each of the OSKM factors from literature (Table 1) and used GenomicLayers to create BindingFactors that would set a layer to *Active* surrounding bound sites according to the *statewidth* parameter (Fig. 1b). All activating binding factors shared the same *statewidth* and *abundance* parameter values. A generic repressor *bindingFactor* was added that would model the accumulation of epigenetic silencing at CpG islands (~ two-thirds of human gene promoters feature a CpG island). In this model, the repressors were set to not bind if there was an activating mark but the activating OSKM factors were allowed to completely ignore the repressive marks. Either or both of these choices could be altered in future models. Simulations ran for 100 cycles on the human hg38 BSgenome (BSgenome.Hsapiens.UCSC.hg38 version 1.4.5). Separate simulations were run varying the *statewidth* parameter shared by all OSKM factors from 50,000 to 200 in irregular intervals (see Table S1). The processing time of the hg38 simulations (Table S1) are an order of magnitude slower than the SacCer3 based simulations and varied as a function of the *statewidth* parameter from ten minutes (statewidth = 50,000) to 204 min (statewidth = 200). Table 1 gives the abundance values of binding factors, which were kept constant throughout. As this model is not included as a Vignette with the GenomicLayers package due to genome size, an R script example version is included as supplemental file 3.

**Table 1:** BindingFactors in the human reprogramming model

BindingFactor	DNA pattern	References	Sites	Abundance
bf_OCT4	ATGCAAAT	[14]	310,995	5000
bf_SOX2	WTTGT	[11]	21,740,984	5000
bf_KLF4	GCCAMGCCTC	[19]	7309	5000
bf_MYC	CACGTG	[12]	571,278	5000
bf_repressor	(CG.{0,20}){4}CG	This study	1,125,634	20,000
bf.RepSpread	NONE	This study	Variable	20,000
bf.cleaner	NONE	This study	Variable	1 × 10^10^

DNA-binding consensus motifs taken from the literature. The “repressor” targets CpG islands by recognising a regular expression including five CpG dinucleotides with zero to 20 other nucleotides between pairs of CpGs. The numbers of potential binding sites in the hg38 genome were determined using the Biostrings R package (used within GenomicLayers). The bf.cleaner is an accessory component to remove all regions in both active and repressed states and is therefore set at an abundance to saturate the genome

To track the behaviour of the model, I measured the proportion of transcription start sites marked ‘active’ or ‘repressed’ during the simulation. To measure if iPSC relevant gene-sets were targeted by the OSKM factors, three gene lists were extracted from a study on the transcriptomic response to OSKM reprogramming from human fibroblasts [33]. The lists (Type I, II, and III) denote distinctively expressed gene-sets in cell sorted populations of cells progressing between fibroblasts and iPSCs. A fourth list was generated including all other genes in the genome. A single transcription start site was chosen for each annotated gene using the MANE select set of transcripts [24] obtained from BioMart (Ensembl Genes 114) using the biomaRt R package (version 2.58.2) [7].

**Fig. 3:**
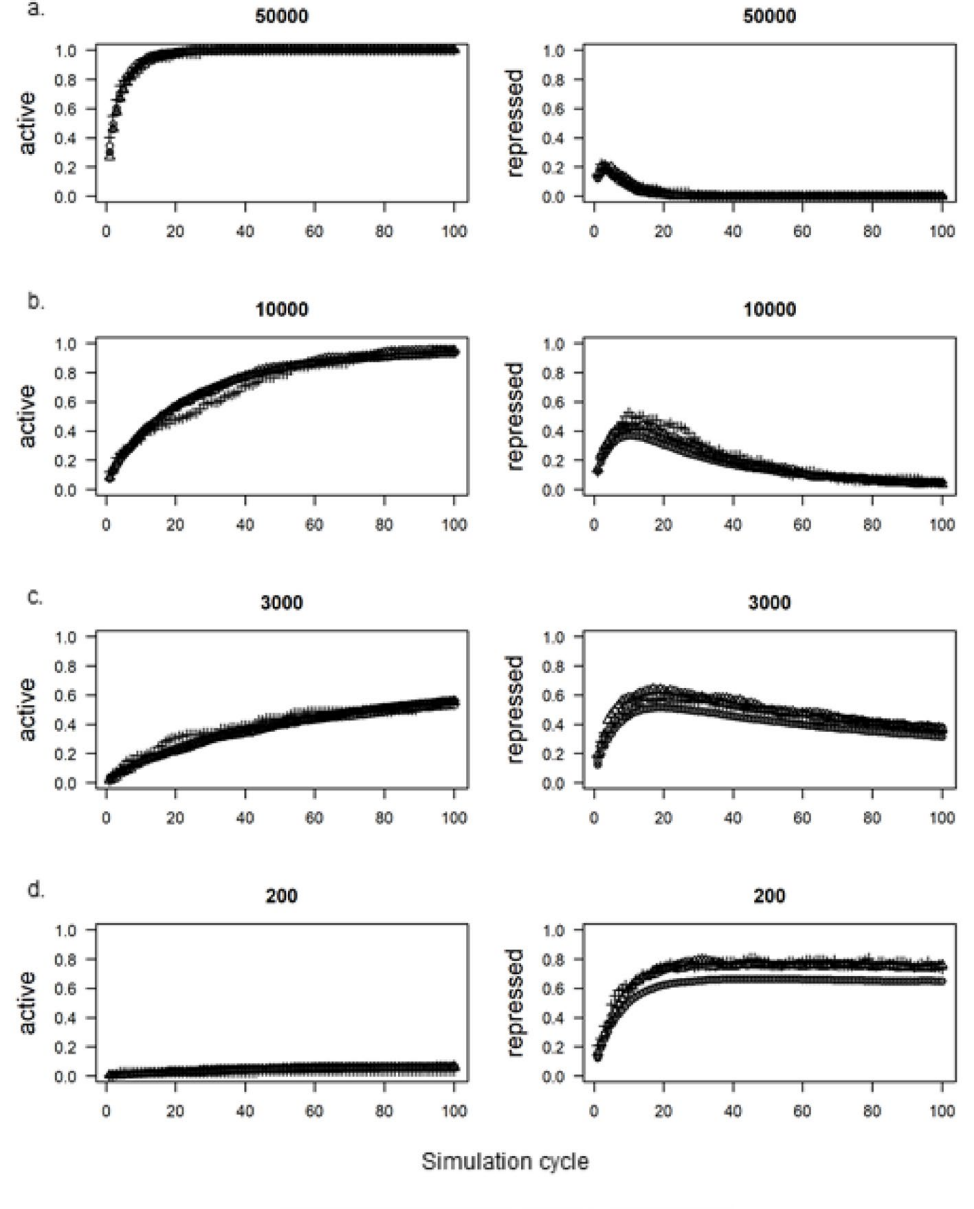
Proportion of Transcription Start Sites (TSS) covered by ‘active’ (left) or ‘repressed’ (right) states during 100 successive simulation cycles. Sub-plots a-d represent separate simulations using decreasing values of the shared statewidth parameter (shown above each plot). The symbols represent proportions from the three gene lists extracted from Tanaka et al. [33]: Open circles: Type I,Crosses: Type II; Open triangles: Type III. Filled grey circles: All other genes

At high statewidth values (20,000 and 50,000), 99% of TSS were marked as ‘active’ by cycle 100 (Fig. 3a, left side; Fig. S2 for all statewidths), which is not surprising as a TSS only need be within half this distance of one of the four OSKM factor motifs to become marked as active. Indeed, 95% of the entire hg38 genome was marked as active by cycle 100 of these simulations (Table S2). Conversely, though up to 20% of TSS in these simulations became repressed early in the simulation (by cycle 5 in Fig. 3a, right side), the OSKM factors over-wrote most of these TSS by the end of the simulation.

At lower statewidth values, the OSKM factors were able only to deposit the ‘active’ state over a minority of TSS within 100 cycles. In these simulations, the repressive factors were able to mark around 60% of TSS in the genome in the same time despite marking no more than 11% of the genome, concordant with the high correlation between CpG islands and gene promoters. Notably, a higher proportion of the OSKM sensitive genes [33] were repressed, which is consistent with the particularly high proportion of CpG islands at promoters of developmentally repressed human genes (see, for example, [10]).

The basic model shown here could be improved by altering either the statewidth or abundance parameters for individual OSKM factors or altering the interaction logic to better match experimental results (e.g. [2]).

## Conclusions and future perspective

GenomicLayers enables rules-based simulations of changes of epigenetic state at genome-wide scale. The models shown here are relatively simples exemplars using the 12 Mb *S. cerevisiae* genome and the 3.2 Gb human genome. The implemented DNA and state-matching allows genomic state, genomic sequence and abundance of “bindingFactors” to interact and cause changes to the state locally and genome-wide. I foresee a range of applications for the software but initially hope that users will follow a two-stage process: (1) run simulations to parameterise and validate rules-based models of genome-wide regulation, informing further experimentation. (2) Share models and components (bindingFactors) to build larger and more complete models. This second goal is inspired by the many overlaps and interactions seen in related epigenetic phenomena as exemplified by mutually exclusive repressive processes of DNA methylation and Polycomb/H3K27me3 silencing in mammals [28].

## Availability and requirements

Project name: GenomicLayers.

Project home page: https://github.com/davetgerrard/GenomicLayers.

Operating system(s): Platform independent.

Programming language: R.

Other requirements: Bioconductor R packages: *BSgenome, GenomicRanges.*

License: GNU GPL3.

Any restrictions to use by non-academics: None.

## Electronic supplementary material

Below is the link to the electronic supplementary material.

Supplementary Material 1. Model output for each nuclear chromosome of SacCer3. See Fig. 2 of main paper for details.

Supplementary Material 2. Model output figure S2 and tables S1 and S2 summarising performance of the hg38 model.

Supplementary Material 3. An R script prototype of the hg38 full human genome model.

## Data Availability

Not applicable.

## Abbreviations

BF: Binding factor
ChIP: Chromatin immuno-precipitation
iPSC: Induced pluripotent stem cell
OSKM: OCT4, SOX2, KLF3, MYC transcription factors
