# Supplementary figures and images for: GenomicLayers: sequence-based simulation of epi-genomes
Source: BMC Bioinformatics. 2025 Aug 4;26:205. doi: 10.1186/s12859-025-06224-y (PMC12323044; doi:10.1186/s12859-025-06224-y)

a)

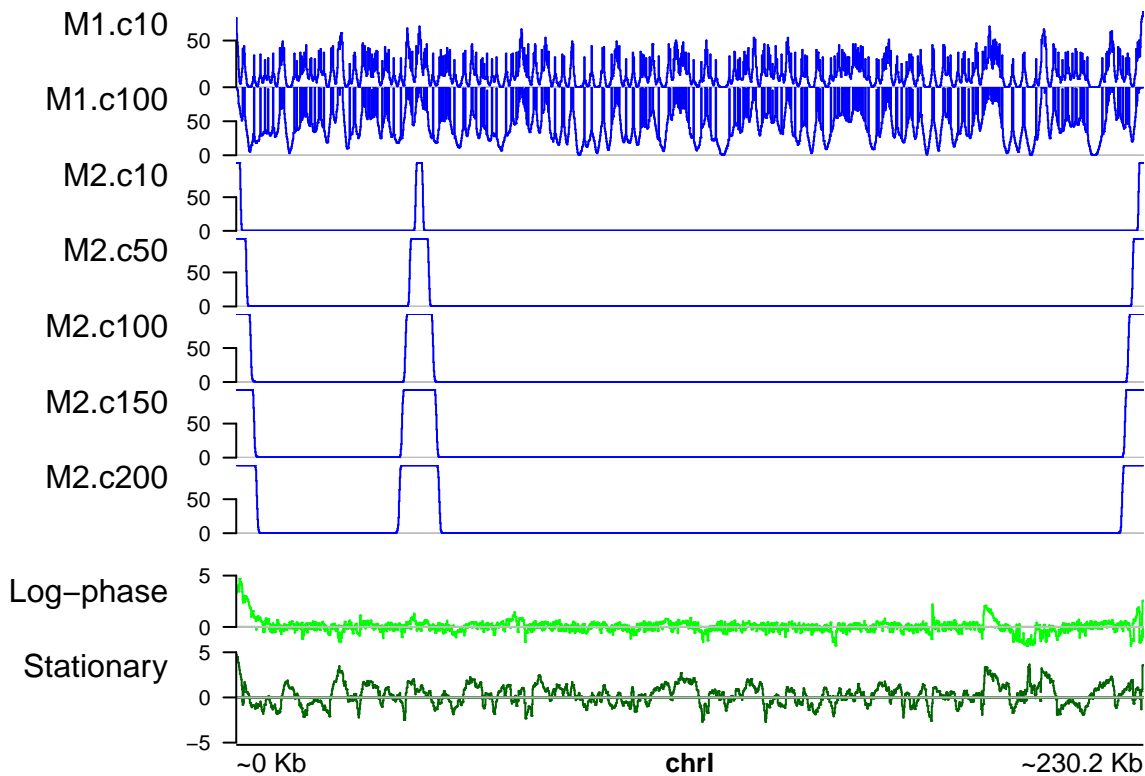

b)

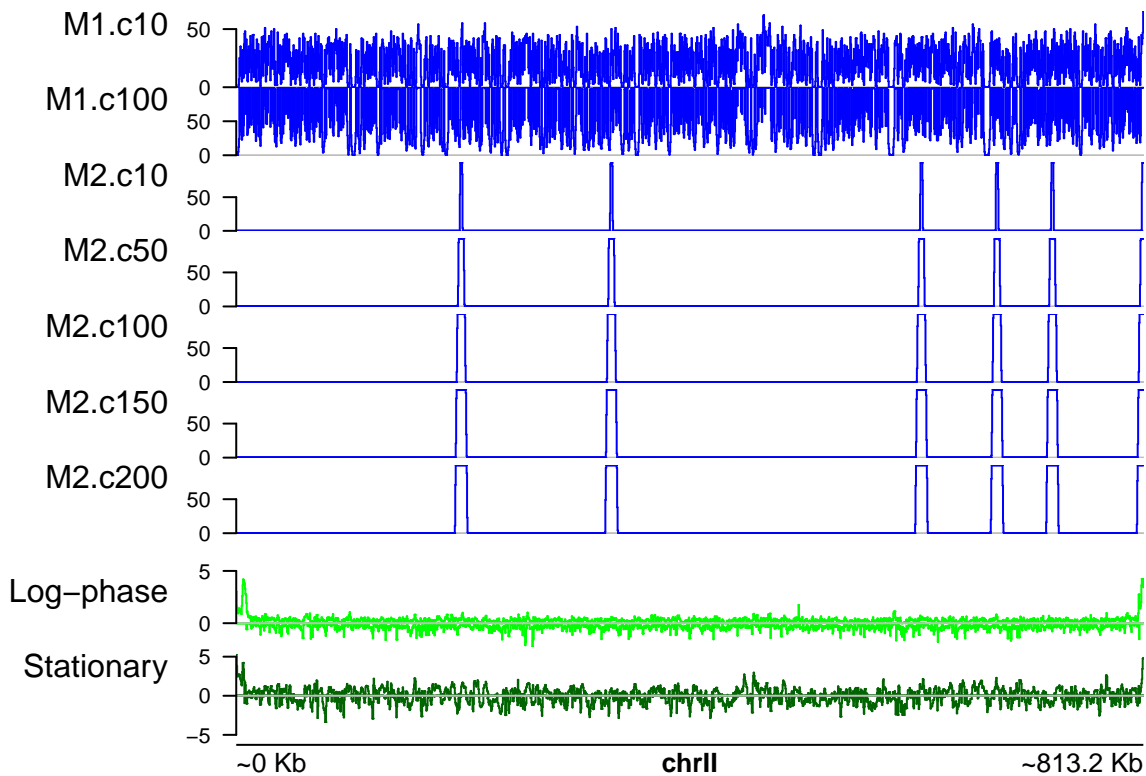

c)

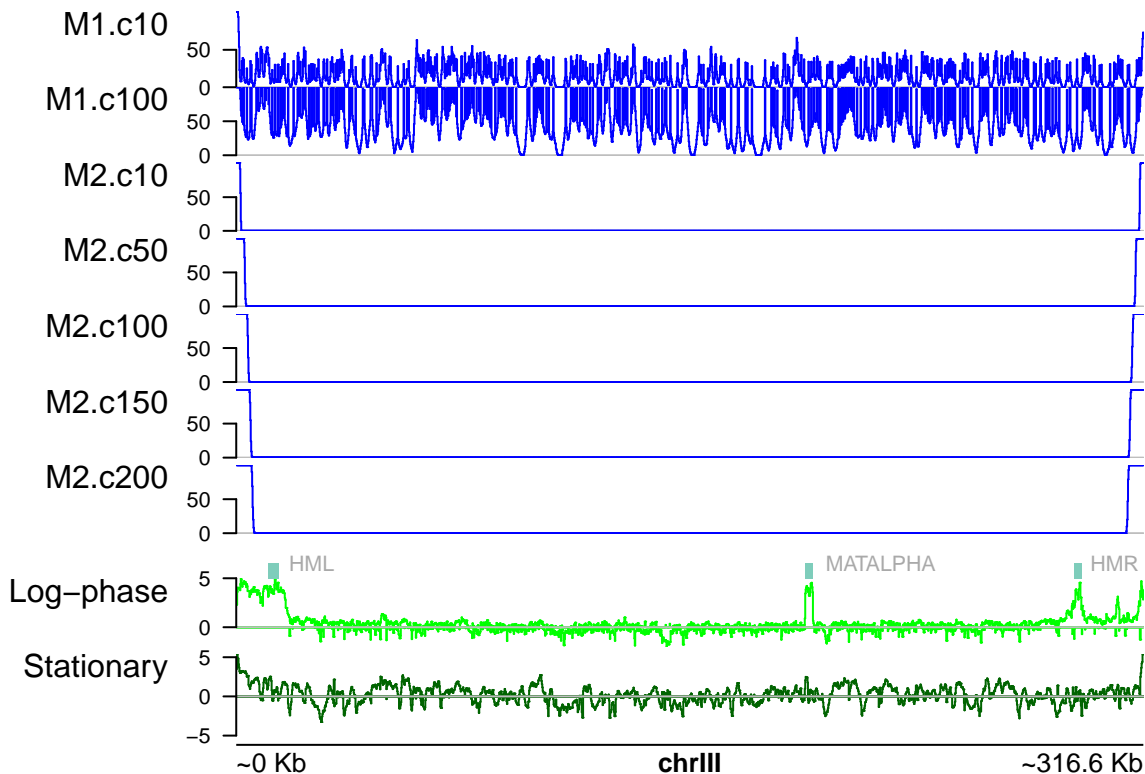

d)

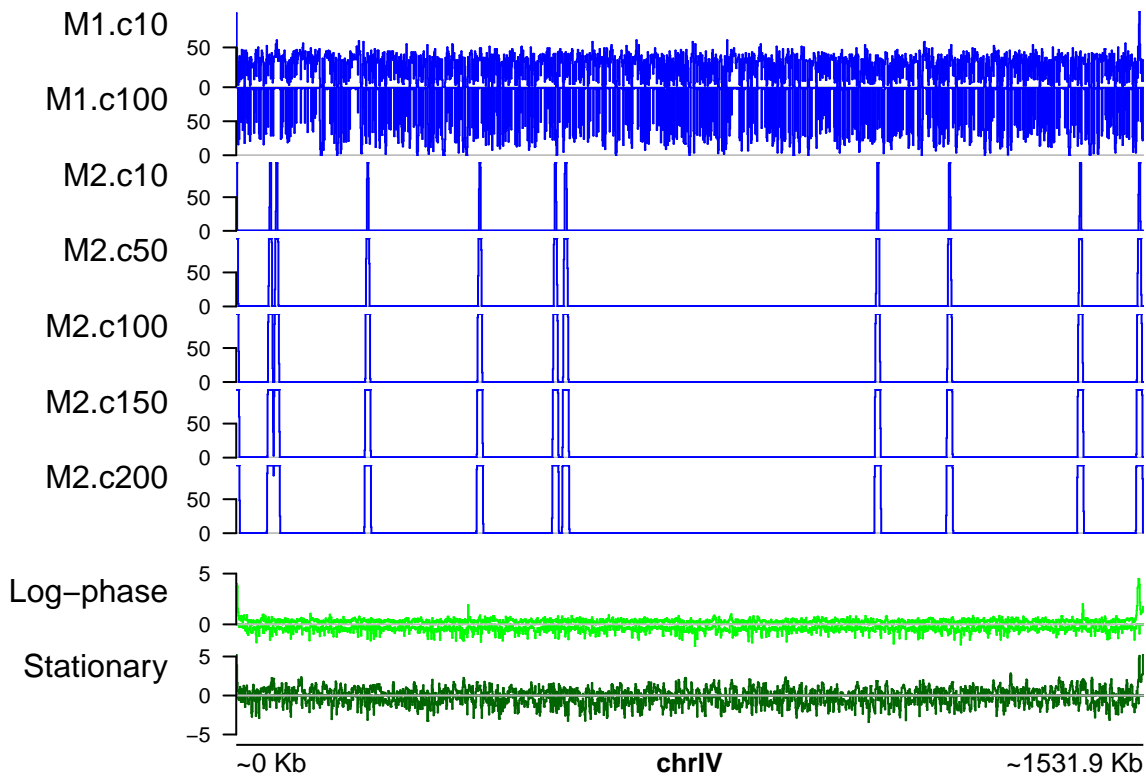

e)

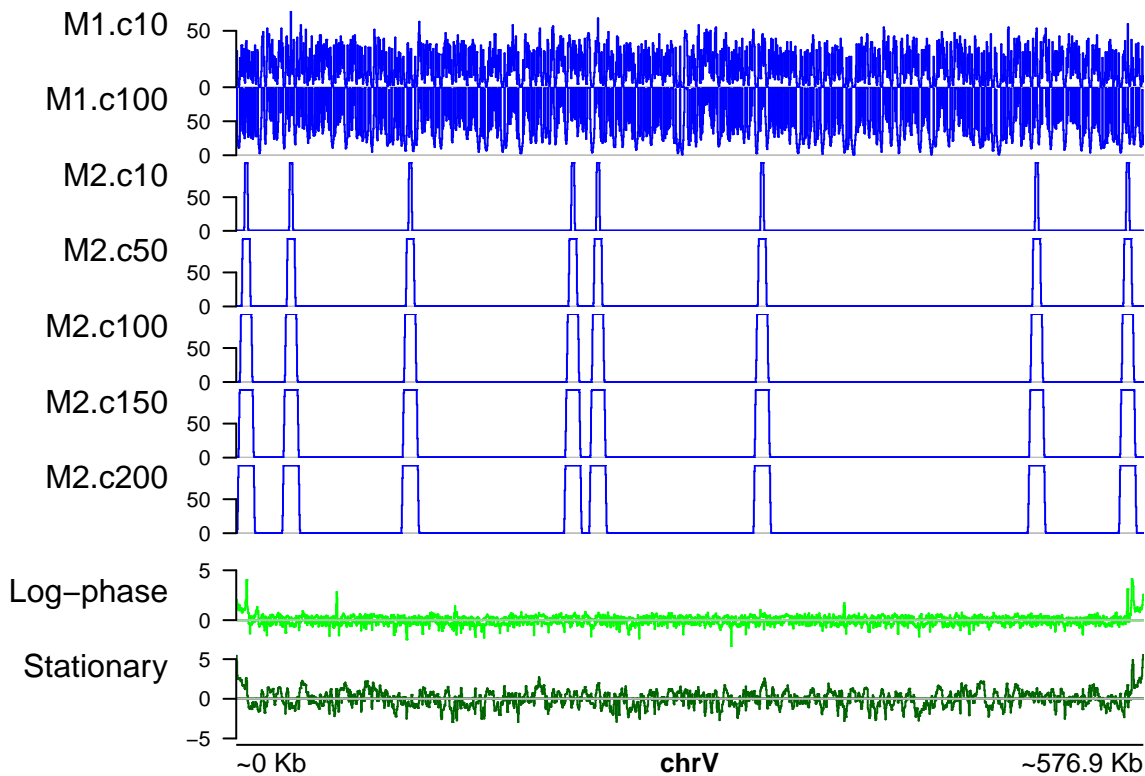

f)

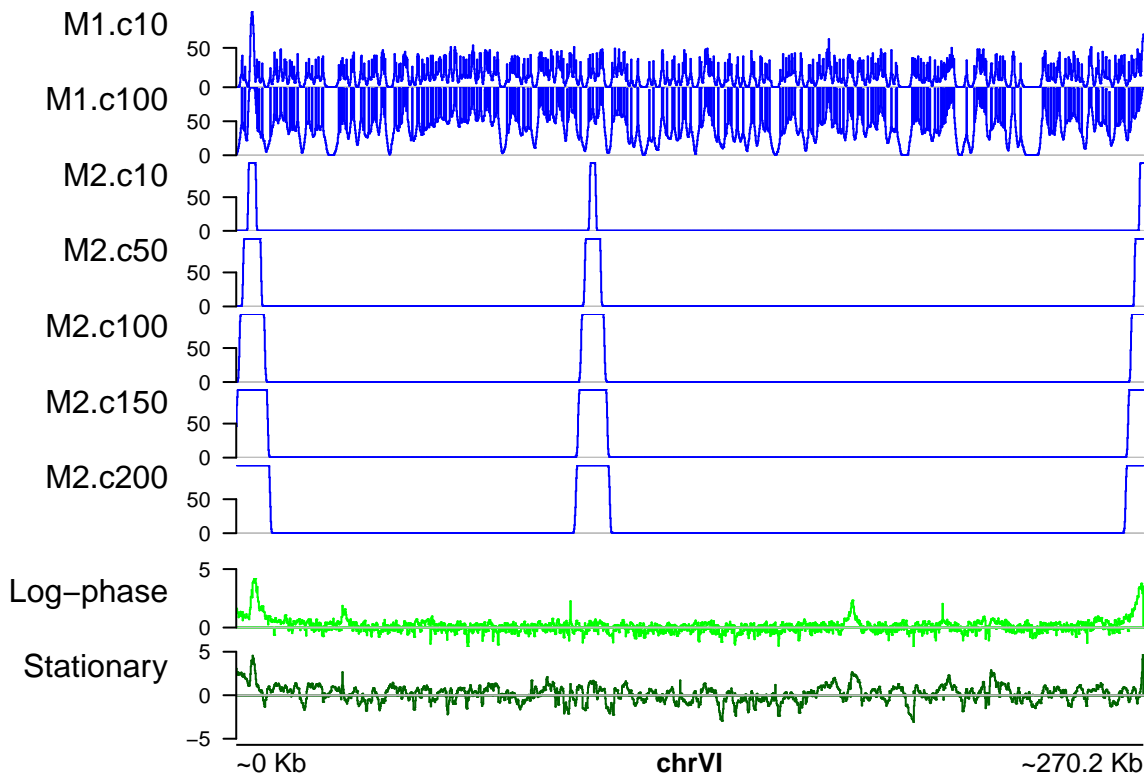

g)

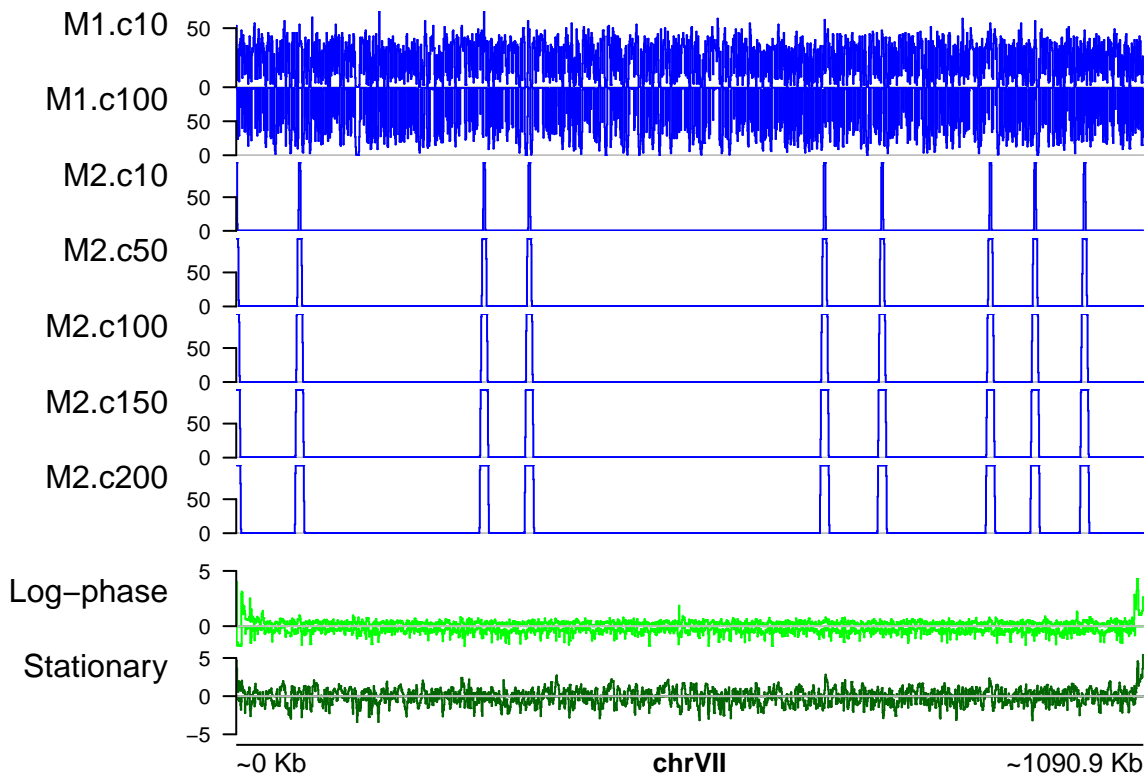

h)

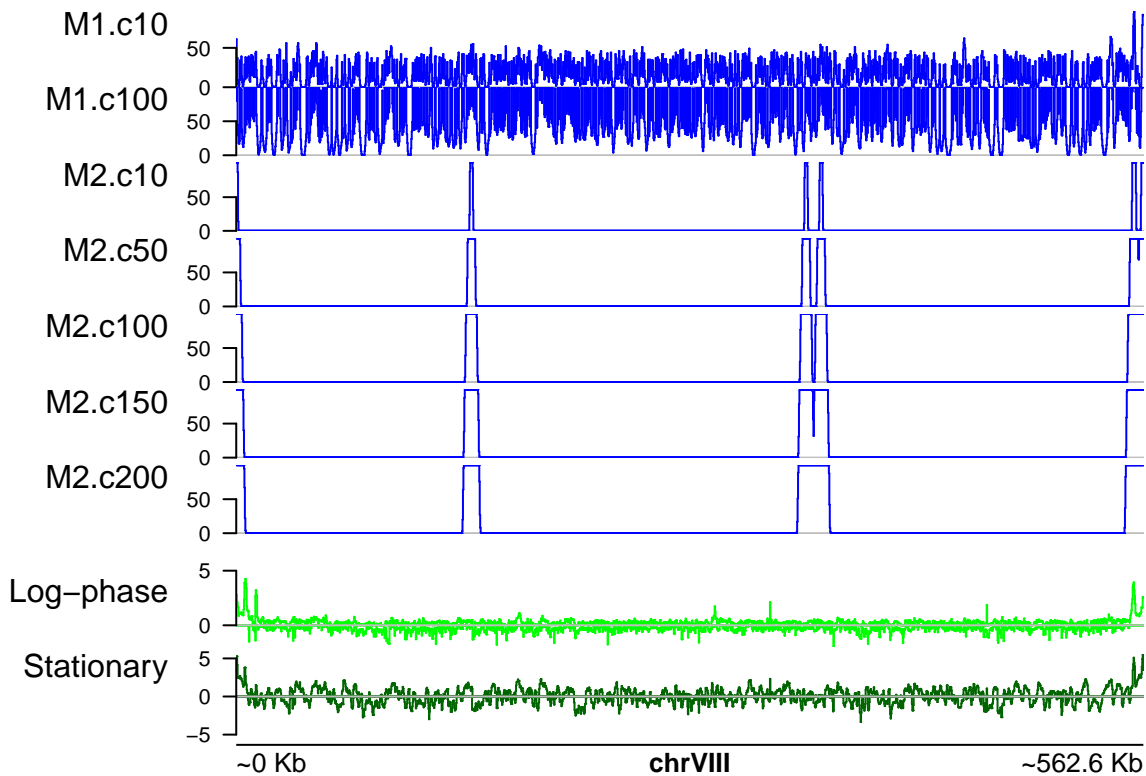

i)

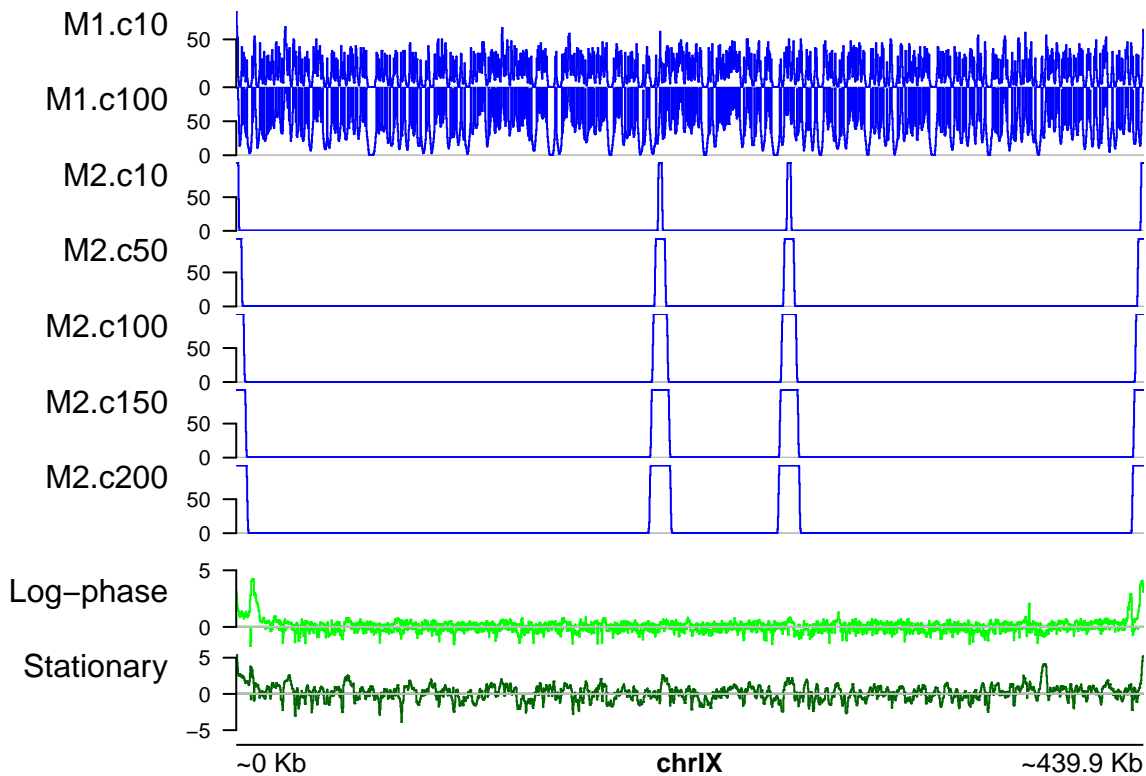

j)

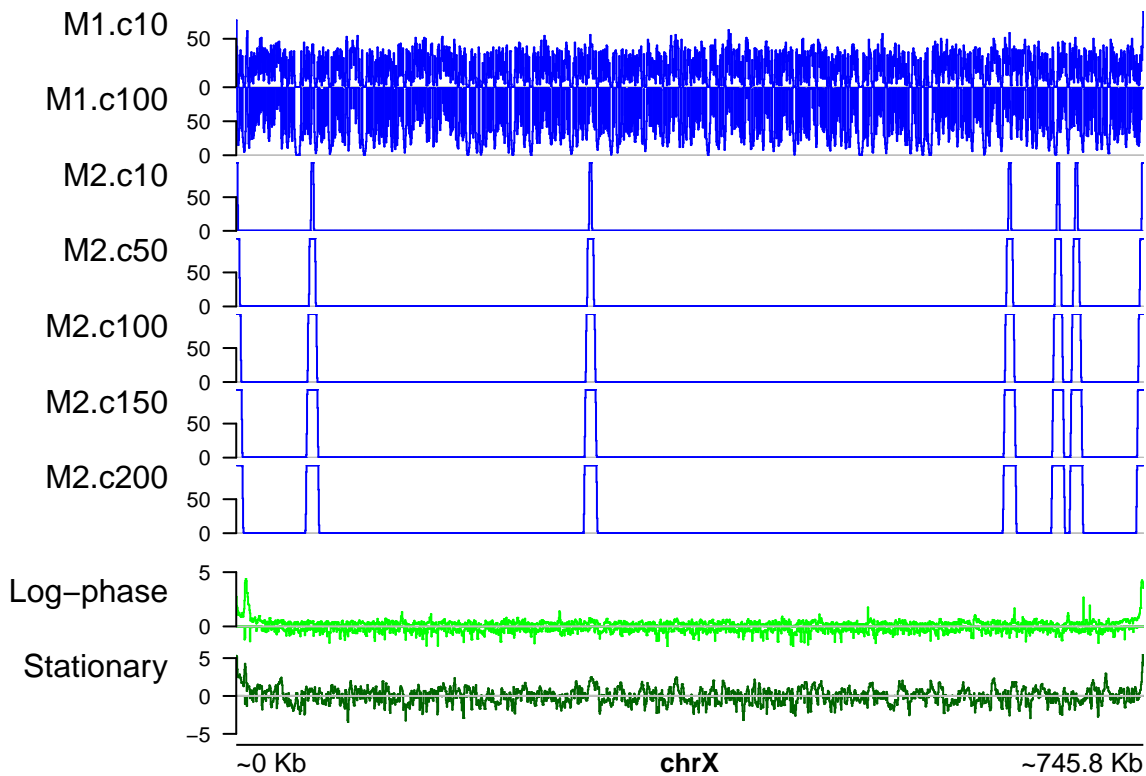

k)

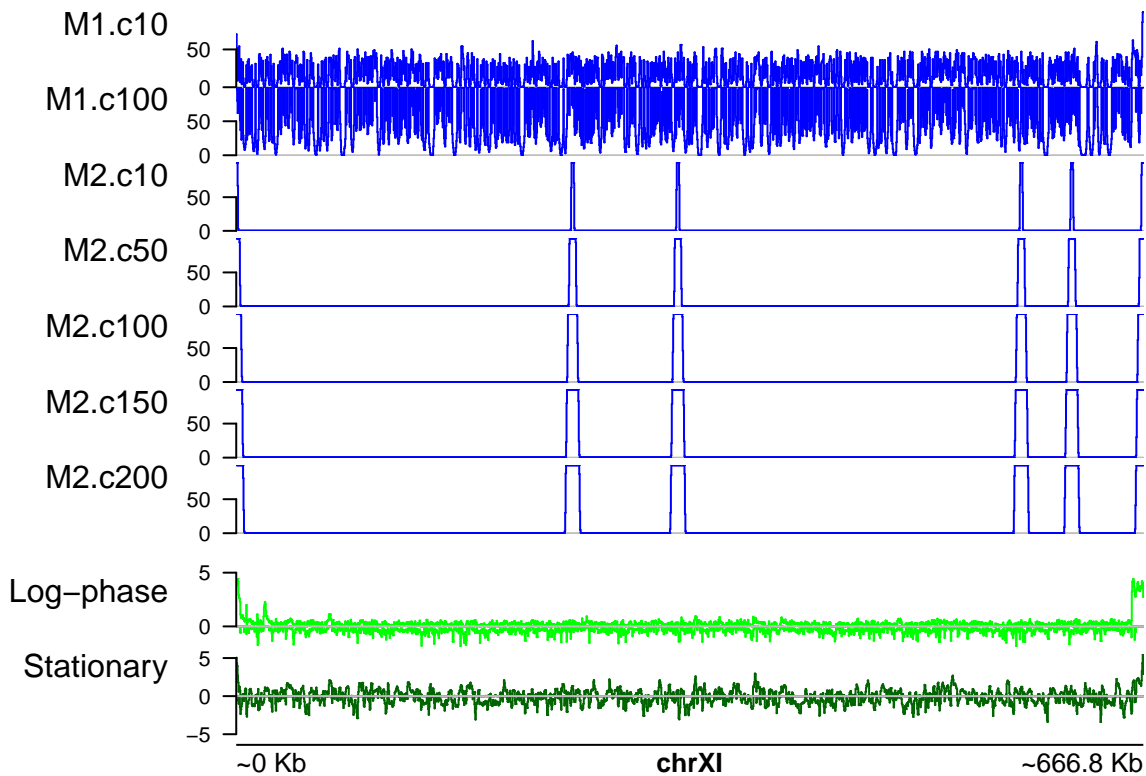

I)

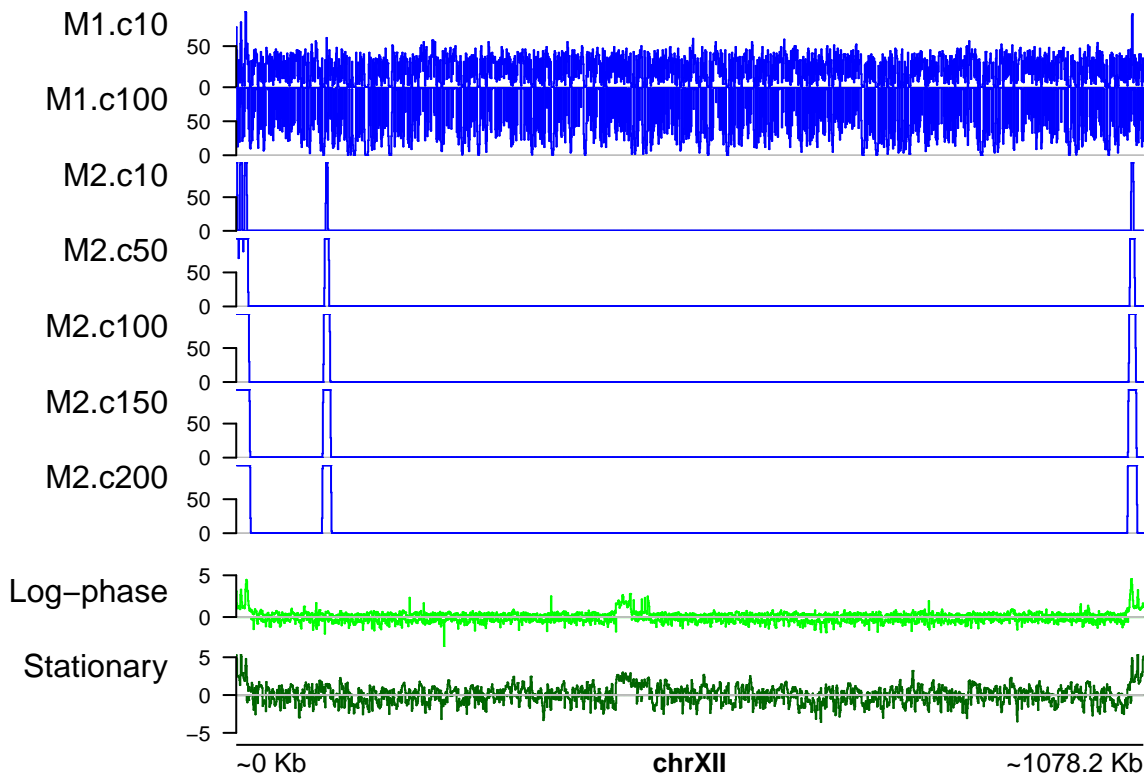

m)

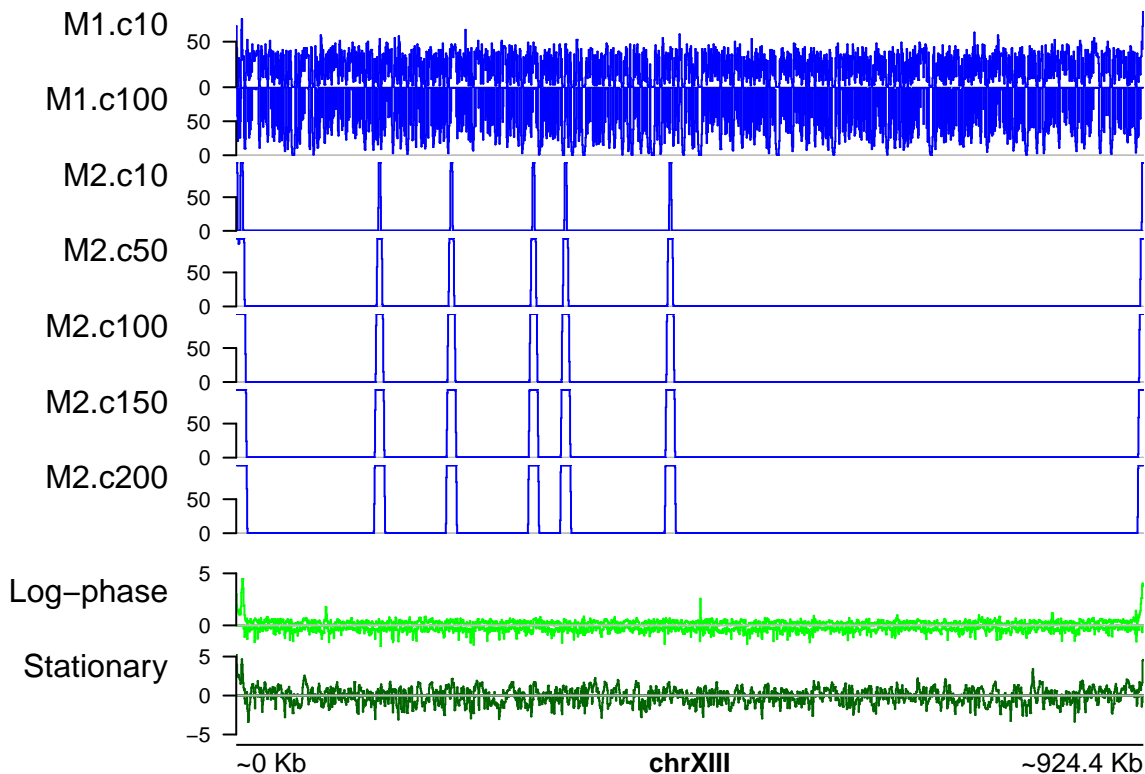

n)

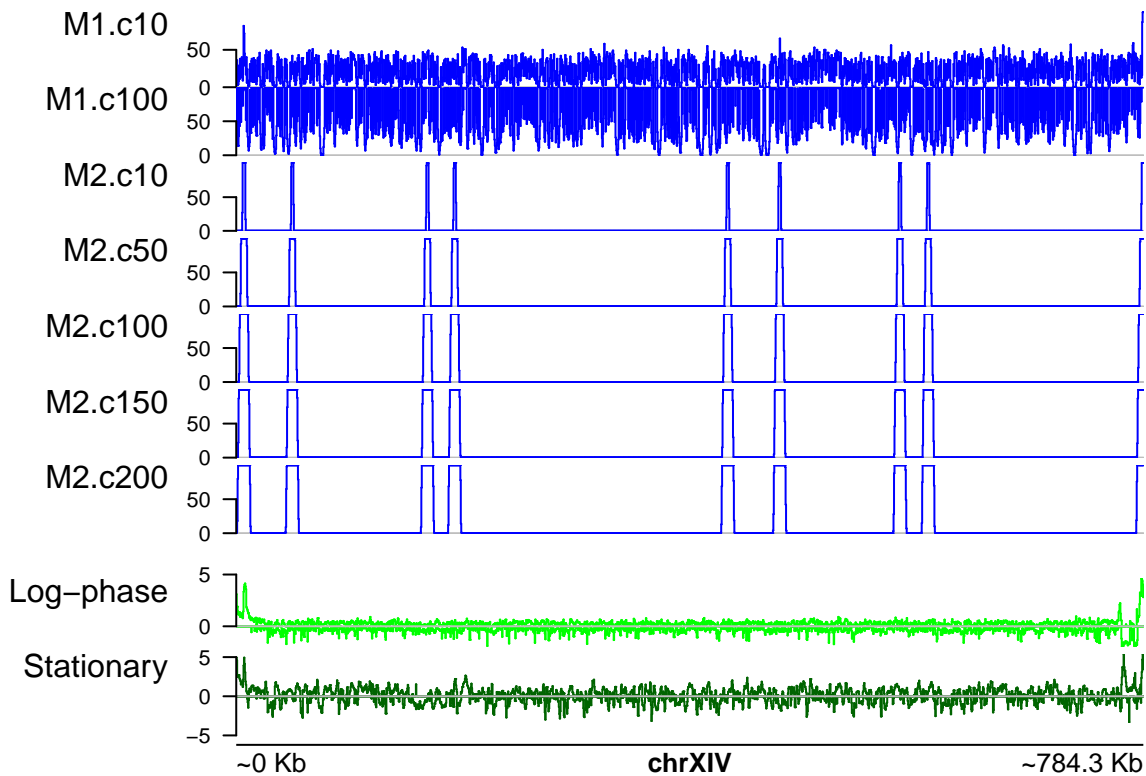

o)

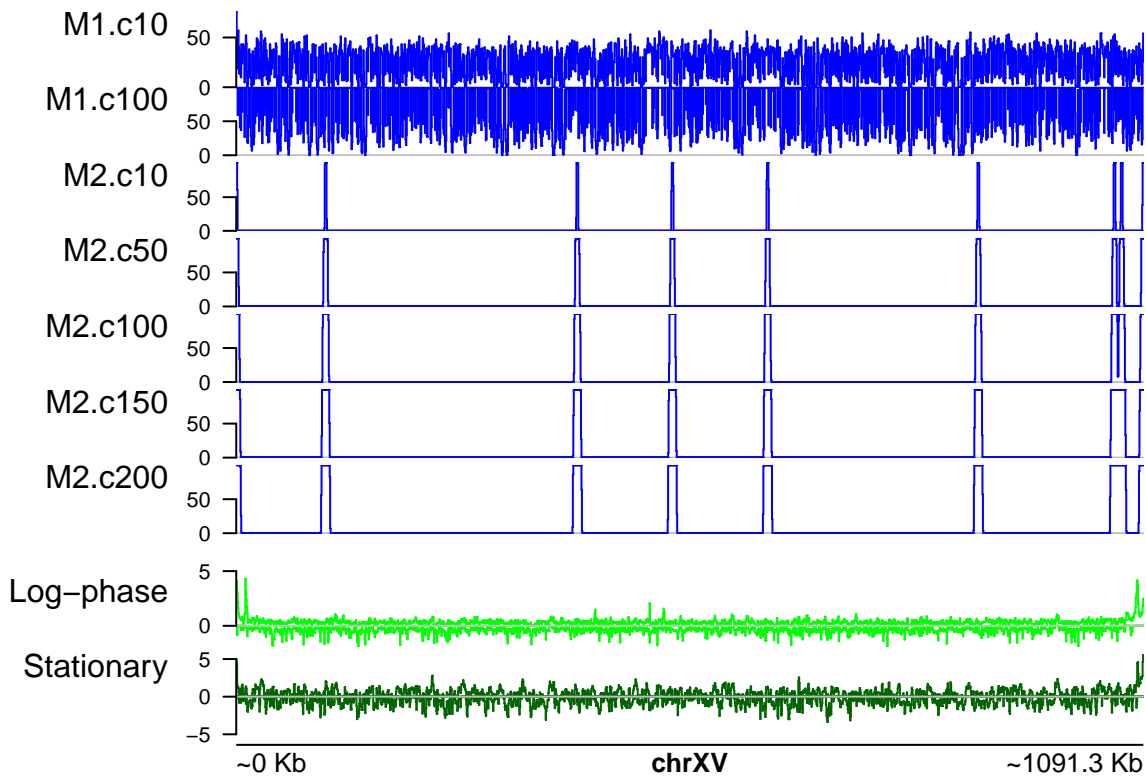

p)

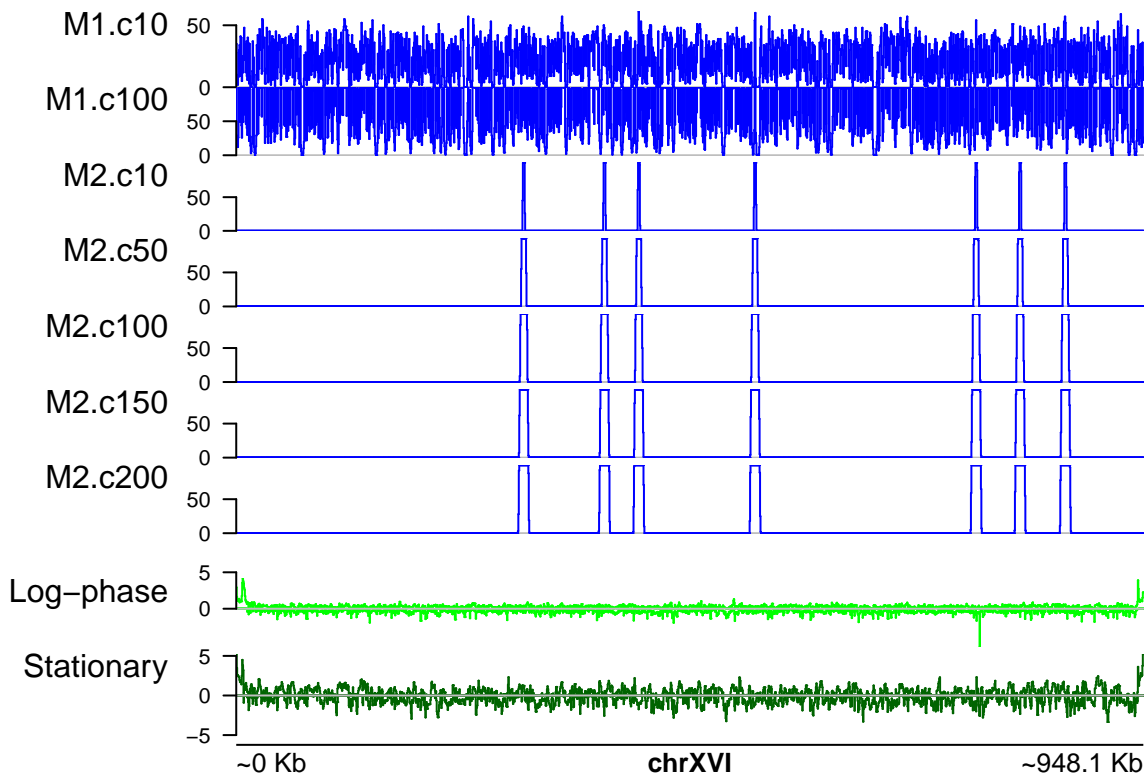

Supplement: Supplementary file 1 — Supplementary Material 1. Model output for each nuclear chromosome of SacCer3. See Fig. 2 of main paper for details. [file 12859_2025_6224_MOESM1_ESM.pdf]
