# Supplementary material for: GenomicLayers: sequence-based simulation of epi-genomes
Source: BMC Bioinformatics. 2025 Aug 4;26:205. doi: 10.1186/s12859-025-06224-y (PMC12323044; doi:10.1186/s12859-025-06224-y)
Supplement: Supplementary file 2 — Supplementary Material 2. Model output figure S2 and tables S1 and S2 summarising performance of the hg38 model. [file 12859_2025_6224_MOESM2_ESM.pdf]

Supplemental file 2

Table S1: Model B (human) run times in minutes for 100 cycles as the statewidth parameter is varied. Mean, minimum and maximum refer to cycle times and total is for the entire simulation (all 100 cycles). Simulations were run using R version 4.3.1 within Rstudio 2024.12 on a Windows 10 PC with an intel CORE i7 processor and 16Gb RAM.

statewidth	mean	min	max	total
50,000	0.10	0.05	0.47	9.61
20,000	0.19	0.07	0.52	18.95
10,000	0.33	0.13	0.41	32.52
3,000	0.93	0.17	1.58	92.07
1,000	1.63	0.12	2.96	161.42
200	2.06	0.12	4.02	203.66

Table S2: % human genome (hg38) coverage in active or repressed states as statewidth parameter is varied. For each state the maximum value obtained and the value after 100 cycles are given.

	Active		Repressed	
statewidth	max	cycle 100	max	cycle 100
50,000	95.4%	95.4%	0.9%	0.0%
20,000	95.0%	95.0%	1.8%	0.1%
10,000	91.2%	91.2%	2.8%	1.3%
3,000	59.5%	59.5%	5.9%	5.9%
1,000	26.9%	26.9%	8.9%	8.9%
200	6.2%	6.2%	10.5%	10.5%

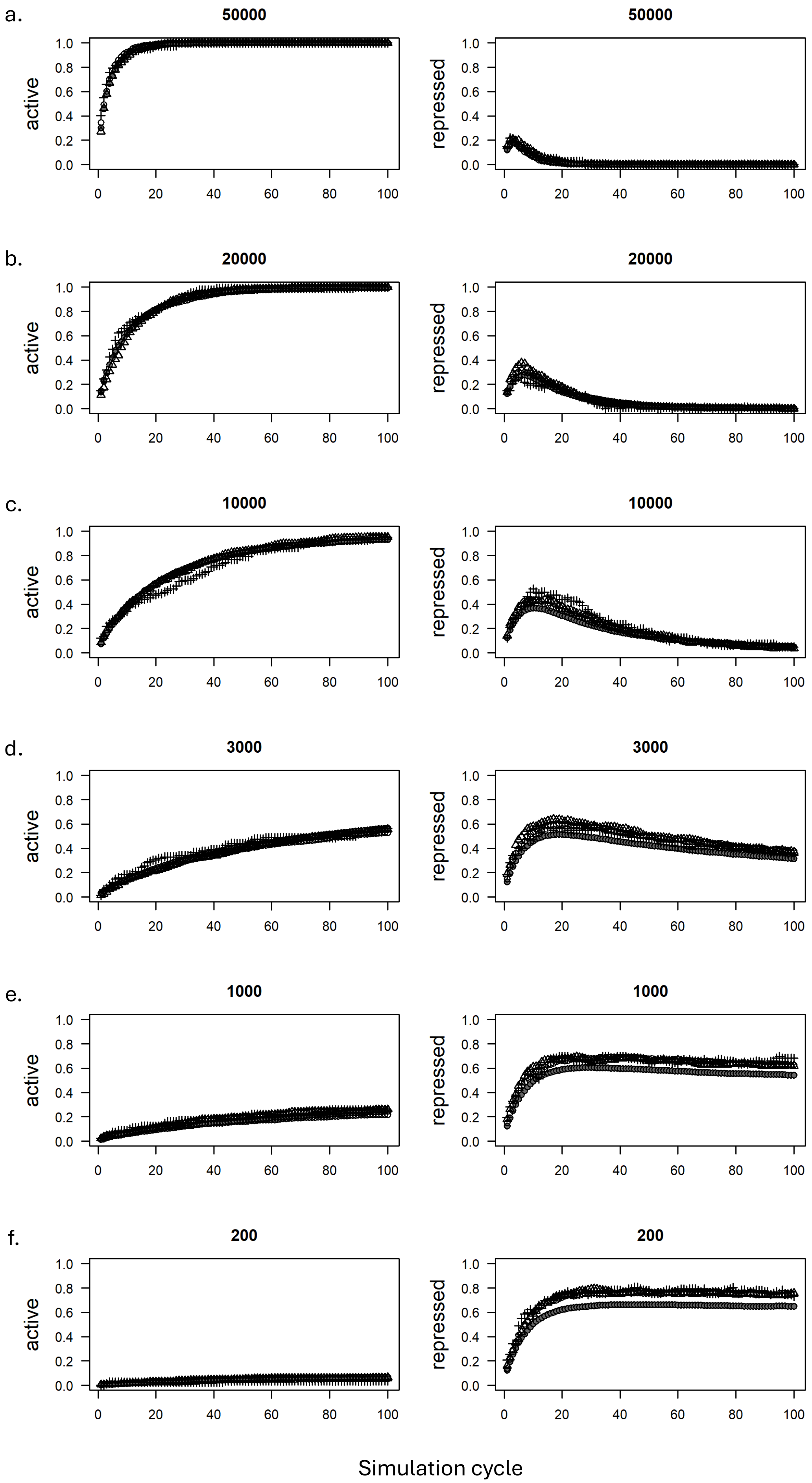
a.
50000
active
repressed
b.
20000
c.
10000
d.
3000
e.
1000
f.
200
0.0
0.2
0.4
0.6
0.8
1.0
0
20
40
60
80
100
Simulation cycle

Figure S2 Proportion of Transcription Start Sites (TSS) covered by ‘active’ (left) or ‘repressed’ (right)states during 100 successive simulation cycles. Sub-plots a-f represent separate simulations using decreasing values of the shared statewidth parameter (shown above each plot). The symbols represent proportions from the three gene lists extracted from Tanaka et al. (2015): Open circles - Type I; Crosses – Type II; Open triangles – Type III. Filled grey circles: All other genes.
